# Transcriptome analysis of two tobacco varieties with contrast resistance to *Meloidogyne incognita* in response to PVY M^S^N^R^ infection

**DOI:** 10.3389/fpls.2023.1213494

**Published:** 2023-08-28

**Authors:** Shixiao Xu, Pei Tian, Zhimin Jiang, Xiaoxiang Chen, Bo Li, Jutao Sun, Zhiqiang Zhang

**Affiliations:** ^1^College of Tobacco Science, Henan Agricultural University, National Tobacco Cultivation & Physiology & Biochemistry Research Centre, Scientific Observation and Experiment Station of Henan, Ministry of Agriculture, Zhengzhou, China; ^2^China Tobacco Jiangsu Industry Co, Ltd. Xuzhou Cigarette Factory, Xuzhou, China; ^3^China Tobacco Zhejiang Industry Co, Ltd., Hangzhou, China

**Keywords:** tobacco, PVY M^S^N^R^, root-knot nematode, transcriptome, resistance

## Abstract

Root-knot nematode (RKN) disease is a major disease of tobacco worldwide, which seriously hinders the improvement of tobacco yield and quality. Obvious veinal necrosis-hypersensitive responses are observed only in RKN-resistant lines infected by Potyvirus Y (PVY) M^S^N^R^, making this an effective approach to screen for RKN-resistant tobacco. RNA-seq analysis, real-time quantitative PCR (qRT-PCR) and functional enrichment analysis were conducted to gain insight into the transcription dynamics difference between G28 (RKN-resistant) and CBH (RKN-susceptible) varieties infected with PVY M^S^N^R^. Results showed that a total of 7900, 10576, 9921, 11530 and 12531 differentially expressed genes (DEGs) were identified between the two varieties at 0, 1, 3, 5, and 7 d after infection, respectively. DEGs were associated with plant hormone signal transduction, starch and sucrose metabolism, phenylpropanoid biosynthesis, and photosynthesis-related metabolic pathways. Additional DEGs related to starch and sucrose metabolism, energy production, and the indole-3-acetic acid signaling pathway were induced in CBH plants after infection. DEGs related to phenylpropanoid biosynthesis, abscisic acid, salicylic acid, brassinosteroids, and jasmonic acid signaling pathway were induced in G28 after infection. Our findings reveal DEGs that may contribute to differences in PVY M^S^N^R^ resistance among tobacco varieties. These results help us to understand the differences in transcriptional dynamics and metabolic processes between RKN-resistant and RKN-susceptible varieties involved in tobacco-PVY M^S^N^R^ interaction.

## Introduction

Biotic stress factors, including phytopathogenic viruses, bacteria, fungi, and nematodes, can infect plants and lead to considerable economic loss (Amorim et al., 2017). Plant growth and yield are seriously affected by plant parasitic nematodes, such as cysts and root-knot nematodes. The host range of root-knot nematode (*Meloidogyne*) species are extensive, and these organisms have been detected in many plant types, including industrial and cereal crops, ornamental plants, vegetables, and weeds (Vos et al., 1998). The major negative effects of infection with *Meloidogyne* are stunted growth, withered leaves, and root knots on the underground portion of plants; the root systems of infected host plants are severely affected, impairing their water and nutrient intake. Plants infected by *Meloidogyne* are also susceptible to infection by other pathogens (Abad et al., 2003).

Tobacco (*Nicotiana tabacum* L.) is an important and widely cultivated industrial crop that can be used to make cigarettes, cigars, chewing or smoking tobaccos, and snuff. Tobacco is also an important model plant for molecular biology research and useful for the study of disease susceptibility in plants of the *Solanaceae* family (Nagata et al., 1992; Sierro et al., 2014). Root-knot nematode (RKN) infection is among the most prevalent diseases of tobacco worldwide and seriously hinders the improvement of tobacco yield and quality. A cost-effective, environmentally friendly, and economical method of controlling RKN damage is to select and promote RKN-resistant varieties; however, laborious phenotyping procedures make it difficult for breeding programs to assess nematode resistance (Giordani et al., 2022), and the identification of RKN-resistant tobacco is the premise and basis for the breeding of RKN-resistant tobacco varieties.

With the rapid development of high-throughput sequencing methods and biotechnology, RKN resistance loci and genes have been identified in some plants (Williamson and Hussey, 1996; Claverie et al., 2011; Barbary et al., 2015; Alekcevetch et al., 2021). Importantly, many RKN resistance genes are pleiotropic; for example, the *Mi* RKN resistance gene of tomato also provides resistance to the potato aphid or whitefly, *Bemisia tabaci* (Dropkin, 1969; Milligan et al., 1998; Rossi et al., 1998; Vos et al., 1998; Nombela et al., 2003). *Gpa2* and *Rx1* are two highly similar *CC-NB-LRR* genes located in the same R gene cluster of potatoes but also confer resistance to two completely different types of pathogens, the potato cyst nematode and Potato virus X (PVX), respectively (Bendahmane et al., 1999; van der Vossen et al., 2000; Slootweg et al., 2017).

Potyvirus is the largest genus of plant viruses that cause significant losses in a wide range of crops. Potato virus Y (PVY) is a species of the potyvirus genus (family, Potyviridae). PVY isolates can be separated into three groups based on the reaction of RKN-resistant and RKN-susceptible tobacco lines to them, as follows: no veinal necrosis-hypersensitive response (HR) is observed in either RKN-resistant or RKN-susceptible lines infected by PVY M^S^M^R^; obvious veinal necrosis-HR is observed in both RKN-resistant and RKN-susceptible lines infected by PVY N^S^N^R^; obvious veinal necrosis-HR is observed only in RKN-resistant lines infected by PVY M^S^N^R^. Further, RKN resistance genes in tobacco are tightly linked to, or pleiotropic with, genes involved in veinal necrosis systemic HR in response to infection by PVY M^S^N^R^. Thus, a rapid leaf assay for HR and necrosis to PVY M^S^N^R^ can be used to screen for RKN-resistant tobacco (Rufty et al., 1987; Fellers et al., 2002; Revers and García, 2015); however, the molecular mechanism underlying this phenomenon remains unclear.

In this study, an RKN-resistant tobacco variety and an RKN-susceptible variety were selected and infected with PVY M^S^N^R^ to analyze the transcriptome dynamics in leaves from the two varieties at different stages after infection. The study objectives were to reveal the underlying molecular mechanisms involved in the veinal necrosis response of RKN-resistant varieties after PVY M^S^N^R^ infection. Our results provide a theoretical basis for screening RKN-resistant varieties through PVY M^S^N^R^ infection and lay a foundation for future breeding of RKN-resistant tobacco.

## Materials and methods

### Plant growth conditions and virus treatment

The tobacco varieties used in this study were CBH (RKN-susceptible) and G28 (RKN-resistant) (Zhu et al., 1989; Li et al., 2010), which were obtained from the breeding laboratory of Henan Agricultural University (Henan, China). PVY M^S^N^R^ was obtained from the Yunnan Academy of Tobacco Agricultural Sciences (Yunnan, China). Tobacco seedlings grown to five or six true leaves were inoculated with PVY M^S^N^R^ solution (comprising 40 ml ddH2O and 1 g leaves of preserved PVY M^S^N^R^ diseased plants), and the leaf surface was lightly rubbed with arenaceous quartz to cause minor wounds. After infection, plants were placed in growth incubators with a photoperiod of 12 h light/12 h dark cycle, light intensity of 1500 lux, with temperatures of 26°C in the light and 22°C in the dark. Tobacco leaves before infection were set as the control group, and tobacco leaves at 1, 3, 5, and 7 days after infection were set as treatment groups; each treatment was repeated three times, and each replicate was prepared by mixing leaves from five plants in each treatment group.

To measure H_2_O_2_, after infection for 7 d with PVY M^S^N^R^, leaves were incubated in 1 mg ml^–1^ diaminobenzidine (DAB) solution (pH 3.8) at room temperature for 8 h. Stained seedlings were then transferred to 70% (v/v) ethanol to remove the chlorophyll. DAB staining was repeated three times.

### RNA extraction, library construction, and sequencing

RNA isolation was performed using the mirVana miRNA Isolation Kit (Ambion, Inc., Austin, TX, USA). To determine the degradation, contamination, concentration, and integrity of RNA, analyses using 1% agarose gels, a Qubit® RNA Assay Kit with a Qubit®2.0 Fluorometer (Life Technologies, CA, USA), and a Bioanalyzer 2100 system (Agilent Technologies, CA, USA) were conducted. Subsequent analysis was performed using samples with an RNA Integrity Number ≥ 7. Library construction was performed using a TruSeq Stranded mRNA LT Sample Prep Kit (Illumina, San Diego, CA, USA). Thirty libraries were sequenced by Shanghai OE Biotech on the Illumina NovaSeq platform (Illumina, San Diego, CA, USA) to generate 150-bp paired-end reads. All sequence data were deposited in the NCBI database (BioProject accession: PRJNA984343).

### Quality control and mapping

Trimmomatic version 0.36 was used to process raw reads (raw data) (Bolger et al., 2014). Low-quality reads and poly-N reads were filtered out, and high-quality, clean reads were finally obtained. Clean reads were mapped to the reference genome using Hisat2 version 2.2.1.0 (Kim et al., 2015).

### DEGs identification and gene function analysis

Differences between G28 and CBH transcriptome data were assessed by principal component analysis (PCA). Cufflinks version 2.2.1 was used to calculate fragments per kilobase per million reads (FPKM) values (Trapnell et al., 2010) and htseq-count to obtain read counts for all genes (Anders et al., 2015). Expression trend analysis was performed using OmicShare Tools (https://www.omicshare.com/tools/Home/Soft/trend); FPKM data for genes in each sample were imported, and the default settings were used. The R package, DESeq version 1.18.0, was used to identify DEGs. Significantly differential expression was defined as P < 0.05 and fold-change > 2 or < 0.5 (Anders and Huber, 2010). The Venn and column diagrams were drawn using TBtools with default settings (Chen et al., 2020). R packages based on the hypergeometric distribution (GO and KEGG) were used for gene function analysis (Kanehisa et al., 2008). The top 20 functional pathways of each group were selected for Heatmaps plotting. The heatmaps of functional analysis were based on Log_10_^(p-value of each pathway)^ through OmicShare Tools with the default settings.

### Validation of RNA sequencing data

The changes in expression of six randomly selected genes were confirmed by conducting qRT-PCR using an ABI 7500 fast Real-Time PCR System (Applied Biosystems, Waltham, MA, USA). Briefly, the total RNAs were isolated as described previously. A TranScript All-in-One First-Strand cDNA Synthesis SuperMix for qPCR Kit (Transgen Biotech, Beijing, China) was employed to synthesize the cDNA. qRT-PCR was performed following the protocol of the TransStart Top Green qPCR SuperMix kit (Transgen Biotech, Beijing, China). The Primer-BLAST online NCBI tool was used to design primers specific for each DEG (**Table S1**). The L25 gene was used as the internal reference to normalize the relative expression levels. The gene expression level of C0 (before infection of CBH) and G0 (before infection of G28) were set to one, and the 2^−ΔΔCt^ method was used to calculate the relative expression level of each assessed gene (Livak and Schmittgen, 2001). Sequencing data were verified by comparing the expression trends of each gene as determined by qRT-PCR and FPKM values. Three biological technical replicates were performed to validate the results of qRT-PCR.

## Results

### Response of tobacco varieties to PVY M^S^N^R^

**Figure 1:**
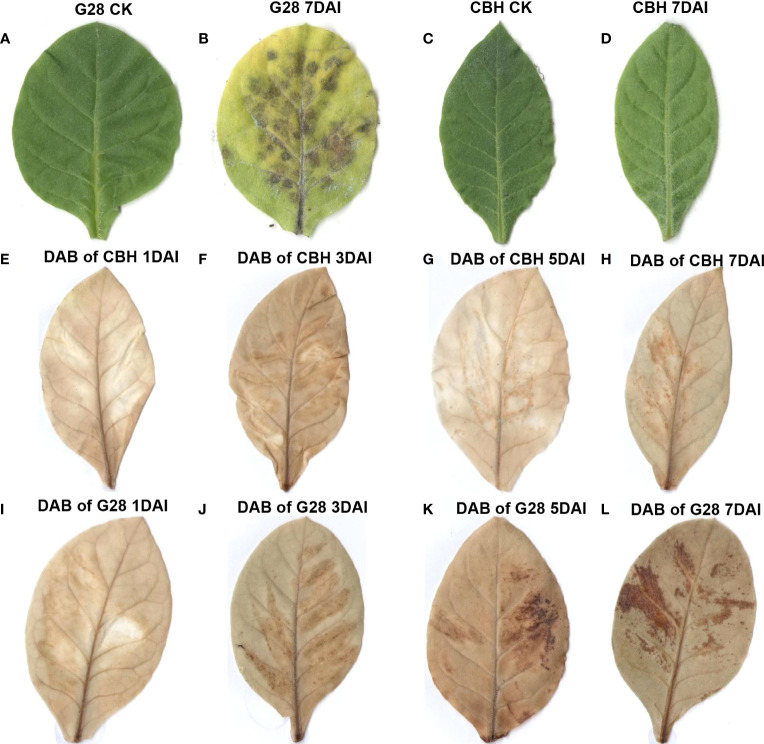
Phenotypic and physiological changes after infection of G28 and CBH tobacco leaves with PVY M^S^N^R^. **(A)** G28 leaf before infection. **(B)** G28 leaf after infection for 7 d. **(C)** CBH leaf before infection. **(D)** CBH leaf after infection for 7 d. **(E–H)** DAB staining of CBH leaves after infection for 1, 3, 5, and 7 d. **(I–L)** DAB staining of G28 leaves after infection for 1, 3, 5, and 7 d.

Leaves of the two tobacco varieties were compared after infection for 7 d with PVY M^S^N^R^. As shown in **Figures 1A–D**, the RKN-resistant variety, G28, developed necrosis spots caused by HR at 7 d, while the RKN-susceptible variety, CBH, did not. Qualitative analysis of hydrogen peroxide in the two varieties after infection was conducted by DAB staining (**Figures 1E–L**). Hydrogen peroxide content did not change significantly in CBH leaves after infection; however, hydrogen peroxide staining was clearly observed in G28 leaves one day after infection and increased and became more diffuse in the following days.

### Overview of RNA-sequencing

**Figure 2:**
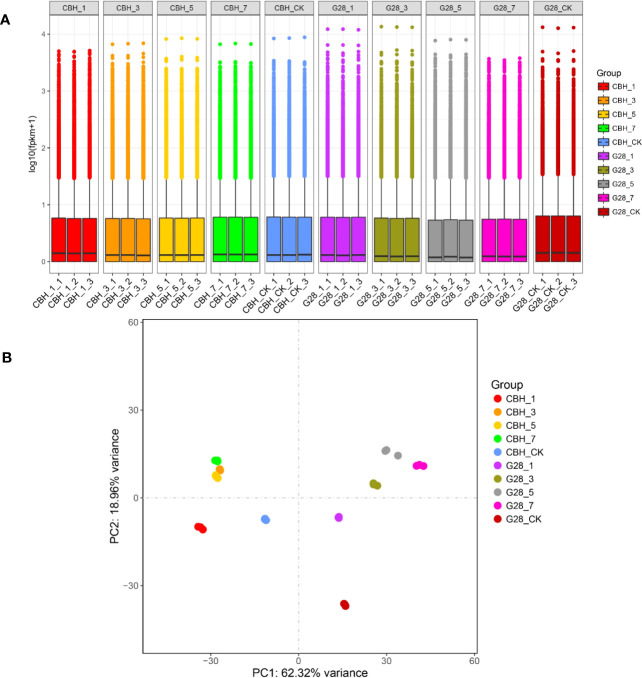
Overview of the transcriptomes of G28 and CBH tobacco varieties after infection with PVY M^S^N^R^. **(A)** Expression density of genes in the two varieties after infection. **(B)** Principal component analysis of genes identified from 30 samples analyzed by RNA-seq.

RNA sequencing data statistics are presented in **Table S2**. The overall gene expression levels of the three biological replicates of each sample were similar, and the identified genes showed differences in expression levels on different days after infection in the two varieties (**Figure 2A**). The repeatability and differentiation of each sample were assessed by PCA analysis. The three replicates of each sample clustered together, and G28 and CBH samples were easy to distinguish on the PC1 axis (**Figure 2B**).

**Figure 3:**
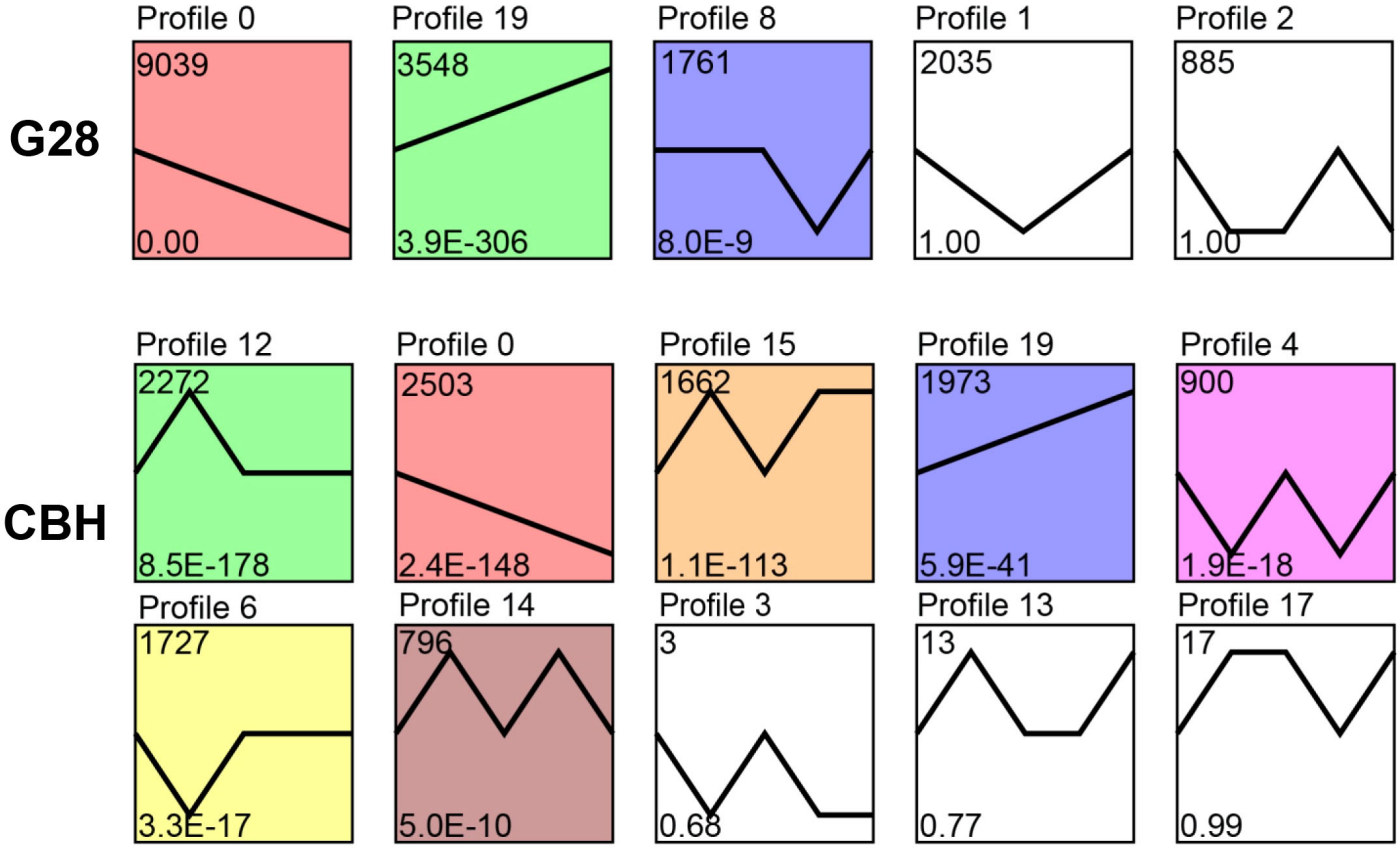
Expression trend analysis of expressed genes after infection of G28 and CBH tobacco varieties with PVY M^S^N^R^. The top number represents the total number of genes in each profile. The bottom number represents the p-value. The line represents the gene expression trends of each profile.

Expression trend analysis showed that three and seven significantly enriched profiles (p < 0.05) were identified in G28 and CBH samples, respectively. Two model profiles (profile 0, downregulated profile; profile 19, upregulated profile) were identified as common to both varieties (**Figure 3**). The potential functions of genes in profiles 0 and 19 in the two varieties are shown in **Figure S1**.

### Identification and analysis of DEGs

**Figure 4:**
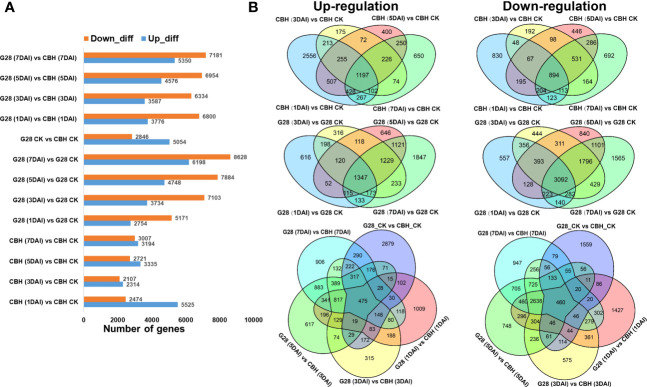
Numbers of specific DEGs in different comparison sets after infection of G28 and CBH tobacco varieties with PVY M^S^N^R^. **(A)** Numbers of up- and down-regulated DEGs. **(B)** Venn diagrams highlighting unique DEGs.

Numbers of DEGs 1, 3, 5, and 7 d after infection, each compared with 0 d, were calculated for both plant varieties. More up-regulated DEGs were found in CBH at each stage, while more genes were suppressed in G28. The number of DEGs between the two varieties at different time points is also presented (**Figure 4A**).

After infection, the number of common upregulated and downregulated DEGs was relatively higher in G28 (1347 and 3092 DEGs, respectively) than in CBH (1197 and 894 DEGs, respectively) at 1, 3, 5, and 7 d compared with normal conditions (**Figure 4B**).

### GO and KEGG analysis of DEGs

**Figure 5:**
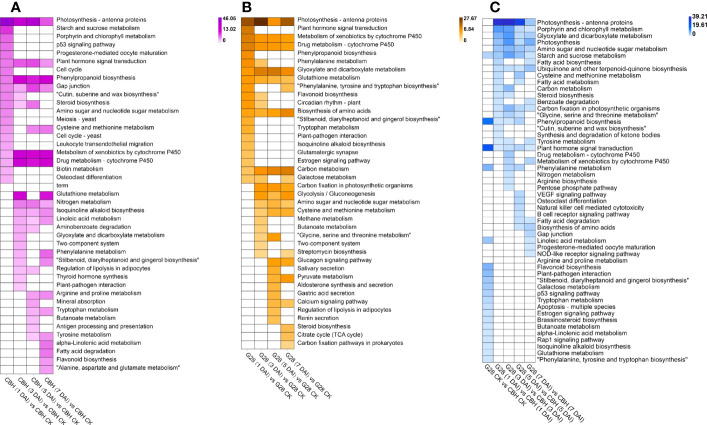
Functional analysis of DEGs in various comparison groups. KEGG pathway analysis of DEGs in **(A)** CBH and **(B)** G28 after 1, 3, 5, and 7 d infection with PVY M^S^N^R^ compared with control samples. **(C)** KEGG pathway analysis of DEGs at each stage in G28 compared with CBH. Changes in p-value are indicated by a change in color. The darker color is used to represent the smaller p-value.

The results of KEGG pathway enrichment analysis for both varieties after infection for 1, 3, 5, and 7 d compared with uninfected controls are shown in **Figure 5**. The ‘Photosynthesis - antenna protein’, ‘Metabolism of xenobiotics by cytochrome P450’, and ‘Drug metabolism - cytochrome P450’ pathways were common to both G28 and CBH varieties after inoculation. Further, ‘Plant hormone signal transduction’ and ‘Phenylpropanoid biosynthesis’ were specifically enriched in CBH after inoculation, while ‘Glyoxylate and dicarboxylate metabolism’, ‘Glutathione metabolism’, ‘Biosynthesis of amino acids’, and ‘Carbon metabolism’ were specifically enriched in G28 after infection. Significant functional differences among DEGs between G28 and CBH at each stage after infection are shown in **Figure 5C**. Photosynthesis-related metabolic pathways (‘Porphyrin and chlorophyll metabolism’, ‘Carbon fixation in photosynthetic organisms’, ‘Photosynthesis’, and ‘Photosynthesis - antenna proteins’), ‘Plant hormone signal transduction’, ‘Starch and sucrose metabolism’, and ‘Phenylpropanoid biosynthesis’ were the main metabolic pathways that differed between the two varieties.

GO enrichment analysis was also performed to identify the putative function of the DEGs after infection (**Figures S2**–**S4**).

### DEGs between infected CBH and G28 related to plant hormone signal transduction

**Figure 6:**
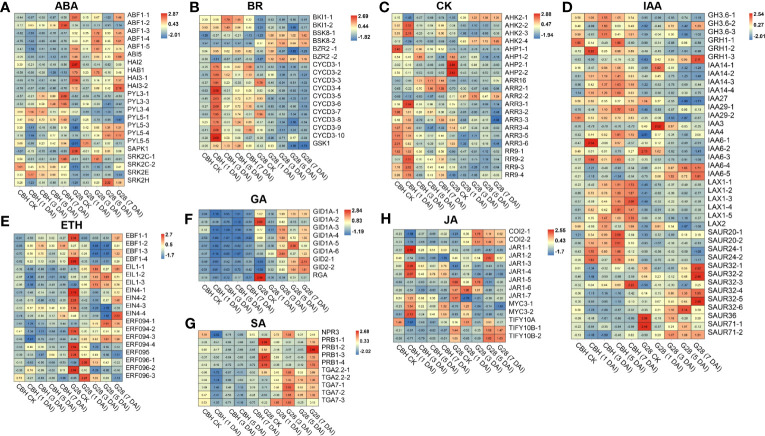
Heatmap analysis of genes associated with **(A)** ABA, **(B)** BR, **(C)** CK, **(D)** IAA, **(E)** ETH, **(F)** GA, **(G)** SA, and **(H)** JA signal transduction after infection of G28 and CBH tobacco varieties with PVY M^S^N^R^. Changes in expression level are indicated by a change in color. Red is used to represent the up-regulation of the genes, and blue is used to represent the down-regulation of the genes.

DEGs involved in abscisic acid (ABA) signal transduction were identified. Further, four and three *PYL* genes were highly expressed in CBH and G28 leaves, respectively. Notably, all identified *ABF*, *ABI*, and *PP2C*-like genes were expressed at higher levels in G28, and the expression levels of *SnRK* genes showed opposite trends in the two varieties (**Figure 6A**). DEGs involved in the brassinosteroid (BR) signal transduction pathway were obtained, of which *CYCD3*, *BKI1*, and one gene were highly expressed in CBH; two *BSK* genes (*BSK8-1* and *BSK8-2*) were highly expressed in G28; and *BZR2-1* was highly expressed in CBH, while *BZR2-2* was highly expressed in G28 (**Figure 6B**). DEGs involved in the cytokinin (CK) signal transduction pathways were obtained; almost all *ARR* genes were highly expressed in CBH (**Figure 6C**). All identified DEGs in the ethylene, gibberellin, and salicylic acid (SA) signal transduction pathways were highly expressed in G28 (**Figures 6D–F**). Moreover, most indole-3-acetic acid (IAA) signal transduction pathway genes were highly expressed in CBH (**Figures 6G, H**).

### DEGs between infected CBH and G28 related to starch and sucrose metabolism

**Figure 7:**
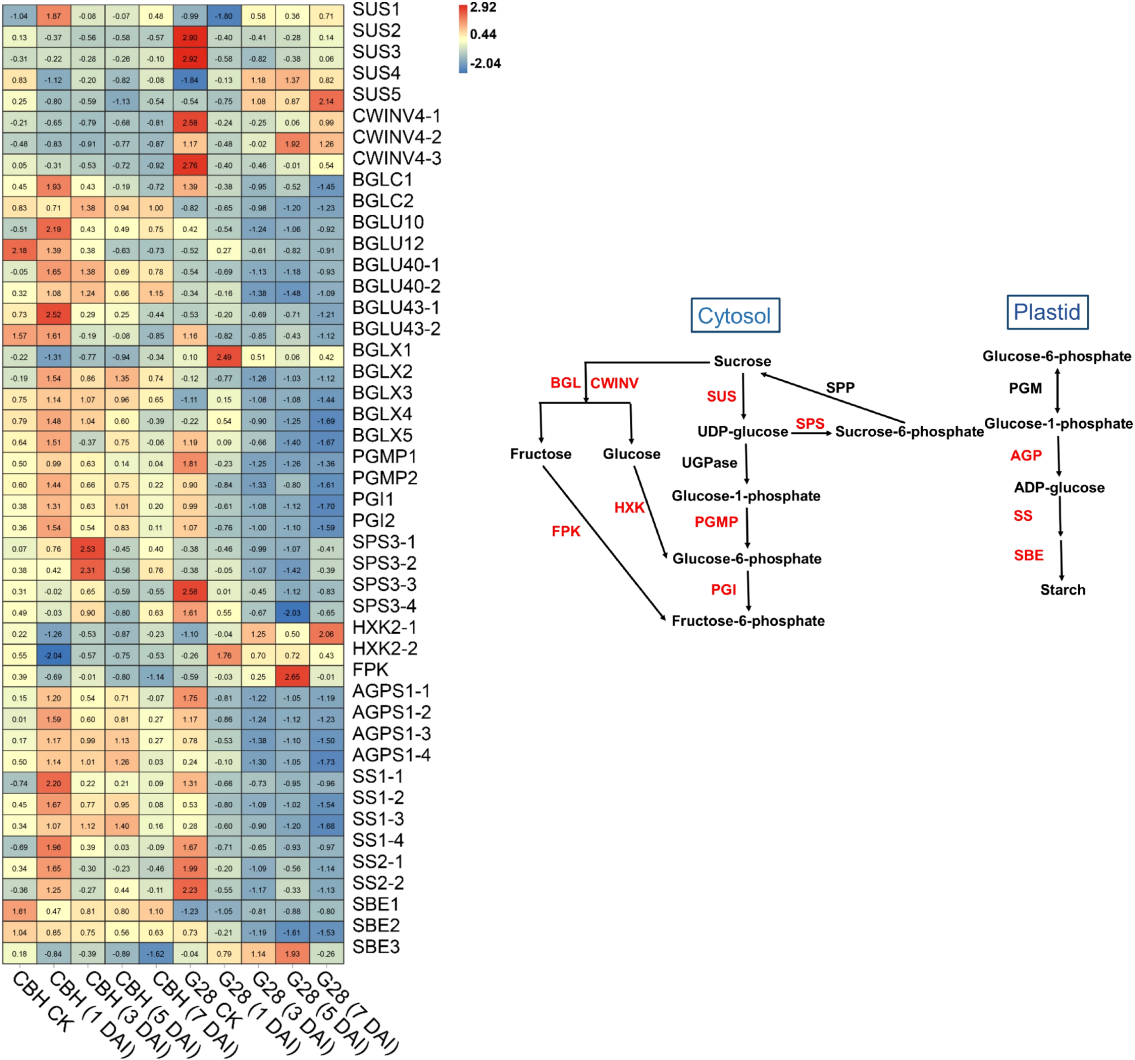
Heatmap analysis of genes associated with starch and sucrose metabolism after infection of G28 and CBH tobacco varieties with PVY M^S^N^R^. Changes in expression level are indicated by a change in color. Red is used to represent the up-regulation of the genes, and blue is used to represent the down-regulation of the genes.

In general, most *SUS* and *CWINV* genes were expressed at higher levels in G28 than in CBH samples (**Figure 7**), while levels of *SUS1*, *SUS4*, and *SUS5* showed the opposite trend in the two varieties. *SUS2*, *SUS3*, *CWINV4-1*, and *CWINV4-3* expressions gradually decreased after infection in both varieties. *CWINV4-2* gene expression also presented a reduced trend in CBH but began to increase at 3 d after infection in G28, reaching a peak at 5 d after infection. The *BGL*, *PGI*, and *PGMP* genes were all highly expressed in CBH relative to G28 leaves. Most *BGL*, *PGI*, and *PGMP* genes showed an increased trend in CBH but decreased continuously in G28. Expression levels of four *SPS* genes presented a reduced trend in G28 leaves but gradually increased and reached peak expression levels at different stages after infection in CBH. Two *HXK* genes (*HXK2-1* and *HXK2-2*) were downregulated after infection in CBH, with minimum levels at 1 d. In contrast, both *HXK* genes were upregulated after infection in G28, with the maximum levels at 7 and 1 d, respectively. One *FPK* gene was downregulated after infection in CBH, with minimum levels at 7 d; however, its expression was upregulated after infection in G28, with the highest levels at 5 d. Four *AGPS* genes were identified and increased after infection in CBH with maximum levels at 1 d (*AGPS1-1*, *1-2*, and *1-3*) and 5 d (*AGPS1-4*). *AGPS* gene levels decreased after infection in G28, with the lowest levels at 3 d (*AGPS1-1* and *1-2*) and 7 d (*AGPS1-3* and *1-4*). Levels of all six *SS* genes were similar to those of *AGPS* in the two varieties. Two *SBE* genes (*SBE1* and *SBE2*) decreased after infection in the two varieties but were expressed at higher levels in CBH than in G28 samples. *SBE3* was also downregulated after infection in CBH but showed an increased trend in G28, with maximum levels at 5 d.

### DEGs between infected CBH and G28 related to phenylpropanoid biosynthesis

**Figure 8:**
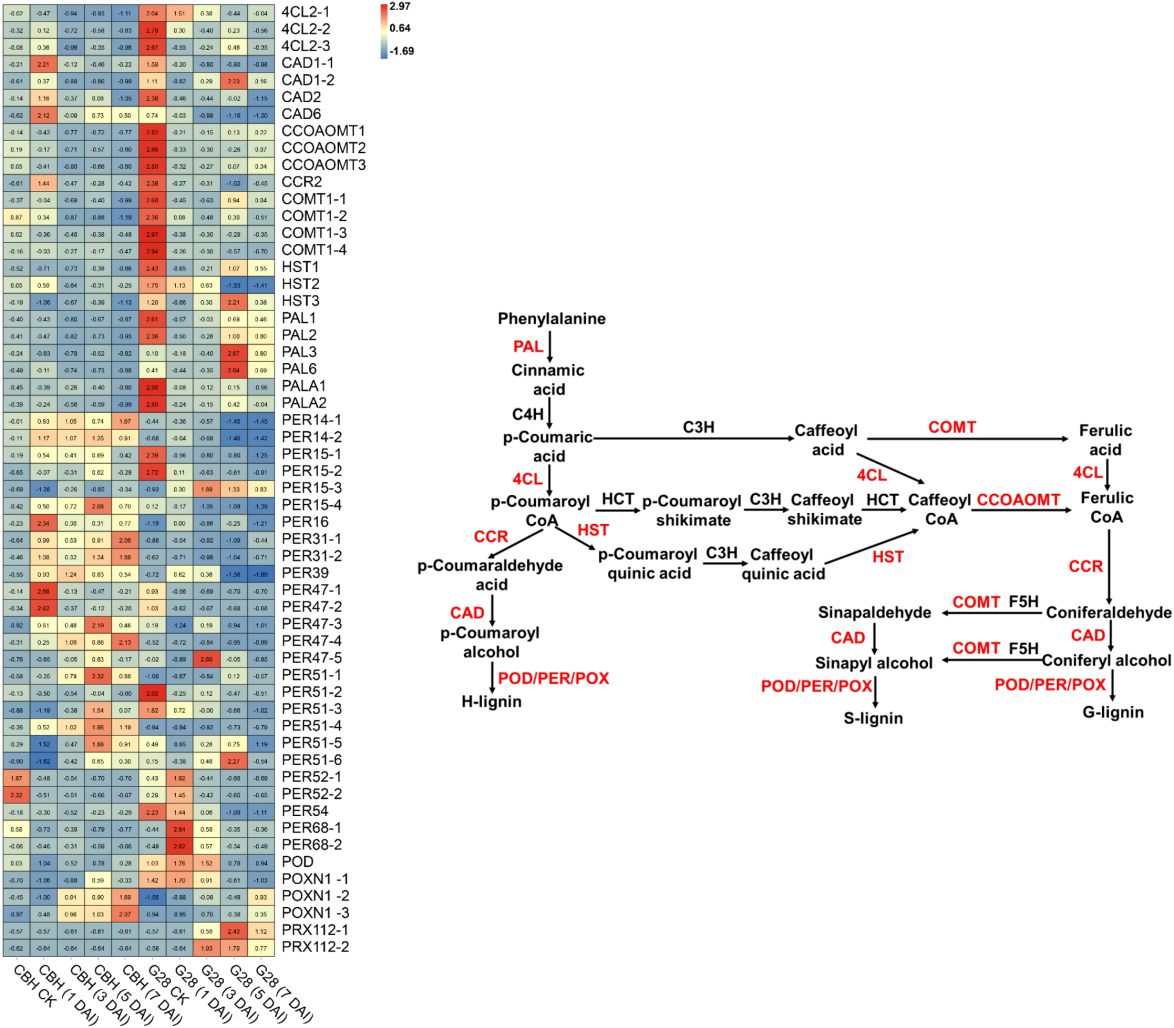
Heatmap analysis of genes associated with phenylpropanoid biosynthesis after infection of G28 and CBH tobacco varieties with PVY M^S^N^R^. Changes in expression level are indicated by a change in color. Red is used to represent the up-regulation of the genes, and blue is used to represent the down-regulation of the genes.

Fifty-six DEGs involved in phenylpropanoid biosynthesis were identified (**Figure 8**). All of the six *PAL* and three *4CL* genes were expressed at higher levels in G28 than in CBH strains, particularly before infection. *PAL* gene expression levels gradually decreased after infection in CBH, while in G28, *PAL1*, *PAL2*, *PALA1*, and *PALA2* presented maximum expression levels before infection, with *PAL3* and *PAL6* reaching peak levels at 5 d. Levels of the *4CL* genes gradually decreased after infection in both CBH and G28 plants. In CBH, four *CAD* genes were induced suddenly after infection and presented maximum expression levels at 1 d, followed by a decline. *CAD* genes gradually decreased after infection in G28, except for *CAD1-2*, which showed the highest expression level at 5 d.

### DEGs between infected CBH and G28 associated with photosynthesis-related metabolic pathways

The levels of DEGs associated with photosynthesis-related metabolic pathways are shown in **Tables S3–S6**. Ninety-one DEGs related to photosynthesis were identified, most of which were induced after infection in CBH, and had maximum expression levels at 1 d, followed by 5 d. These genes were expressed at lower levels in G28 after infection, with minimum levels from 3 to 7 d after infection. Sixty DEGs related to antenna proteins were identified. Most genes were reduced at 1 d, then increased at 3 d after infection and reached peak levels at 5 or 7 d in CBH. In G28, levels of these genes decreased significantly after infection and remained low until 7 d. Seventy-three DEGs involved in carbon fixation in photosynthetic organisms were obtained, of which most levels were reduced after infection in CBH and decreased significantly after infection in G28. Fifty-one DEGs related to porphyrin and chlorophyll metabolism were identified; the majority were induced after infection and reached maximum levels at 1 d, then declined in CBH. In G28, expression of these genes was significantly reduced after infection and remained low for 7 days.

### Verification of RNA-seq data by qRT-PCR

Six DEGs were randomly selected for validation of sequencing data by qRT-PCR. The qRT-PCR assay results exhibited general and high consistency with the sequencing data, indicating that our RNA-seq analysis findings are reliable (**Figure S5**).

## Discussion

Due to the pleiotropy of RKN resistance genes or their tight linkage with PVY M^S^N^R^ resistance genes, it is established that only RKN-resistant tobacco develops obvious veinal necrosis-HR after infection by PVY M^S^N^R^; however, the underlying molecular mechanisms are poorly understood. HR occurs when plants are infected with microbial pathogens, and infection typically results in rapid cell death near the infection site. A series of defense reactions between the initial infection site and surrounding cells are initiated to restrict pathogen growth and represent a form of programmed cell death (PCD) (Heath, 2020; Lam et al., 2021). The resistance of many crops to virus and RKN is closely related to HR induced by reactive oxygen species (ROS) accumulation (Milligan et al., 1998; Melillo et al., 2006; Oliveira et al., 2012). In this study, the RKN-resistant variety, G28, generated necrosis spots caused by HR at 7 d, while the RKN-susceptible variety, CBH, did not. Furthermore, hydrogen peroxide content increased and diffused in G28 in the days after infection, as shown by DAB staining, but did not significantly increase in CBH leaves.

The gene expression and metabolic differences between G28 and CBH induced by PVY M^S^N^R^ were studied using high-throughput sequencing. In G28, the numbers of identified DEGs increased gradually after infection, suggesting that gene expression was more significantly affected as the infection persisted, while this trend was not observed in CBH leaves. The KEGG analysis showed that photosynthesis- related metabolic pathways, plant hormone signal transduction, starch and sucrose metabolism, and phenylpropanoid biosynthesis were common differential metabolic pathways altered in the two varieties at all stages after infection.

During infection, microbes attempt to obtain sugar from plant species, causing the plant to require more nutrients (Berger et al., 2007; Xiao et al., 2022); therefore, plant carbohydrate allocation and signaling pathways are significantly changed following microbial infection (Bezrutczyk et al., 2018). Genes involved in sugar metabolism were induced in a susceptible variety of sugarcane after infection with the sugarcane mosaic virus, while only a few genes were upregulated in resistant varieties; some genes related to starch synthesis were specifically upregulated in the susceptible variety (Akbar et al., 2021). In our study, more DEGs related to starch and sucrose metabolism were induced in CBH leaves after infection. In addition, we found that DEGs related to energy production (photosynthesis) were markedly induced at different stages after infection in CBH, indicating that the virus may manipulate plant energy generation and metabolism for its own needs in this susceptible variety. We hypothesize that G28 resistance to PVY M^S^N^R^ may be due to the limitation of energy production through necrosis spots caused by HR, leading to inhibited virus diffusion.

The phenylpropanoid pathway contributes to plant responses to biotic stressors, such as viruses, fungi, and root-knot nematodes (Li et al., 2017; Behr et al., 2020; Kavil et al., 2021; Arraes et al., 2022). *PAL* is considered a chemical marker of induced resistance in many plants; for example, *PAL1* expression improved disease resistance to cassava brown streak virus in cassava (Kavil et al., 2021). Further, in *Brachypodium distachyon*, *PAL* can promote antiviral defenses against the *panicum mosaic virus* and its satellites (Pant et al., 2021). In the present study, most DEGs related to the phenylpropanoid pathway were expressed at higher levels in G28 than in CBH. In addition, two *PAL* genes (*PAL3* and *PAL6*) were specifically induced in G28 samples, indicating that *PAL* genes may play a key role in resistance to PVY M^S^N^R^.

Phytohormones have been extensively investigated for their role in response to viral infection. Four hormones, including ABA, SA, BR, and JA, have positive effects on plant defenses against biotic stress. ABA stimulates resistance to antiviral diseases; hence, treatment with ABA can increase resistance against viruses (Mauch-Mani and Mauch, 2005; Alazem et al., 2014). In *abi4* mutants, a higher accumulation of TMV-cg was observed (Chen et al., 2013). Cell-to-cell movement of viruses is inhibited by *PP2C* in soybean (Seo et al., 2014). The expression of ABA signaling pathway-related genes is higher in RKN-resistant materials, which may be closely associated with eggplant resistance to RKN (Zhang et al., 2021). In our study, the expression level of *ABI5* and *PP2C*-induced protein (*HAI2*, *HAB1*, *HAI3-1*, and *HAI3-2*) were increased after infection in G28; nevertheless, CBH did not alter the expression of these genes significantly. The SA signaling pathway is important for limiting viral spread at infection sites through increasing ROS and pathogenesis-related proteins and by triggering HR and PCD (Dinesh-Kumar et al., 2000; Baebler et al., 2014). Endogenous SA also plays an important role in RKN resistance (Li et al., 2018; Zhang et al., 2023). Some SA signaling pathway genes, such as *NPR*, *PRB*, and *TGA*, were significantly induced in G28 leaves after infection but showed a downward trend in CBH samples in this study. BRs can enhance plant resistance to viruses (Zhang et al., 2015). In this study, *BSK* and *BZR* expression levels were strongly induced in G28 samples after infection. The JA signaling pathway can also enhance resistance to biotic stress. SlWRKY45 attenuates RKN-regulated JA biosynthesis and represses defense against RKN (Huang et al., 2022). *COI1* knockdown accelerated the development of symptoms and accumulation of viral particles during the early stages of infection (Garcia-Marcos et al., 2013). After infection with PVY M^S^N^R^, *COI* expression levels were specifically induced in G28 leaves. In plants, viruses interact directly with auxin/IAA proteins through the helicase domain to enhance virus phloem loading and accumulation, resulting in abnormal plant development (Jay et al., 2011; Collum et al., 2016). Consistent with this finding, our results indicated that more auxin-related genes were induced after infection in CBH samples. The findings of research on the role of ethylene in plant virus resistance have been inconclusive. In some plant-pathogen interactions, ethylene increases plant resistance, while in others it promotes viral infection (Santner et al., 2009; Zhu et al., 2014; Zhao et al., 2017). In the present study, almost all DEGs related to ethylene signaling were induced in G28 after infection, while no significant changes were observed in CBH samples, indicating that ethylene also has a positive effect on the defense of tobacco against PVY M^S^N^R^. CK positively modulates SA signaling and contributes to virus resistance in plants (Choi et al., 2011). In our study, the expression of CK signaling pathway-related genes differed from that previously reported, in that most genes in the CK signaling pathway were induced in the susceptible variety, CBH, indicating that CK may have negative effects on PVY M^S^N^R^ resistance. Together, the results described above indicate that the functions of phytohormone signaling pathways in response to viral infection are complex and involve interactions among multiple hormones.

## Conclusion

In summary, the RKN-resistant variety, G28, developed an obvious veinal necrosis-HR after infection with PVY M^S^N^R^ to prevent the spread of the virus. This resistance process may be achieved through the following regulatory processes: (1) expression of genes associated with energy and carbohydrate metabolism was down-regulated in G28 compared to CBH after infection, limiting further spread of biotic stress; (2) higher expression of genes involved in phenylpropanoid biosynthesis, particularly *PAL*, in G28 enhances its resistance to biotic stress; (3) induced expression of genes related to ABA, SA, BR, and JA signaling pathways had positive effects on defense of G28 against biotic stress. This study provides a new understanding of the molecular mechanisms involved in veinal necrosis responses of RKN-resistant varieties after PVY M^S^N^R^ infection. Moreover, PVY M^S^N^R^ infection is a useful tool for screening RKN-resistant varieties and also provides a theoretical basis for RKN-resistant tobacco breeding.

## Data availability statement

The datasets presented in this study can be found in online repositories. The names of the repository/repositories and accession number(s) can be found in the article/**Supplementary Material**.

## Author contributions

SX and ZZ conceived and designed the experiments. SX, PT, and ZJ performed the experiments. XC and BL contributed reagents, materials, and analysis tools. SX, JS, and ZZ wrote and revised the manuscript. All authors contributed to the article and approved the submitted version.

## Supplementary material

Supplementary Figure 1 KEGG analysis of genes in profile 0 and profile 19 of G28 and CBH.

Supplementary Figure 2 GO enrichment analysis of DEGs in CBH after infection at 1, 3, 5, and 7 d compared with normal conditions.

Supplementary Figure 3 GO enrichment analysis of DEGs in G28 after infection at 1, 3, 5, and 7 d compared with normal conditions.

Supplementary Figure 4 GO enrichment analysis of DEGs between G28 and CBH at each stage after infection.

Supplementary Figure 5 Quantitative real-time PCR (qRT-PCR) validation and RNA-seq data. Data shown were the mean of three independent repeated experiments ± standard deviation. Error bars represent standard deviations from three independent biological replicates.
