# Supplementary figures and images for: Transcriptome analysis of two tobacco varieties with contrast resistance to Meloidogyne incognita in response to PVY MSNR infection
Source: Front Plant Sci. 2023 Aug 28;14:1213494. doi: 10.3389/fpls.2023.1213494 (PMC10493397; doi:10.3389/fpls.2023.1213494)

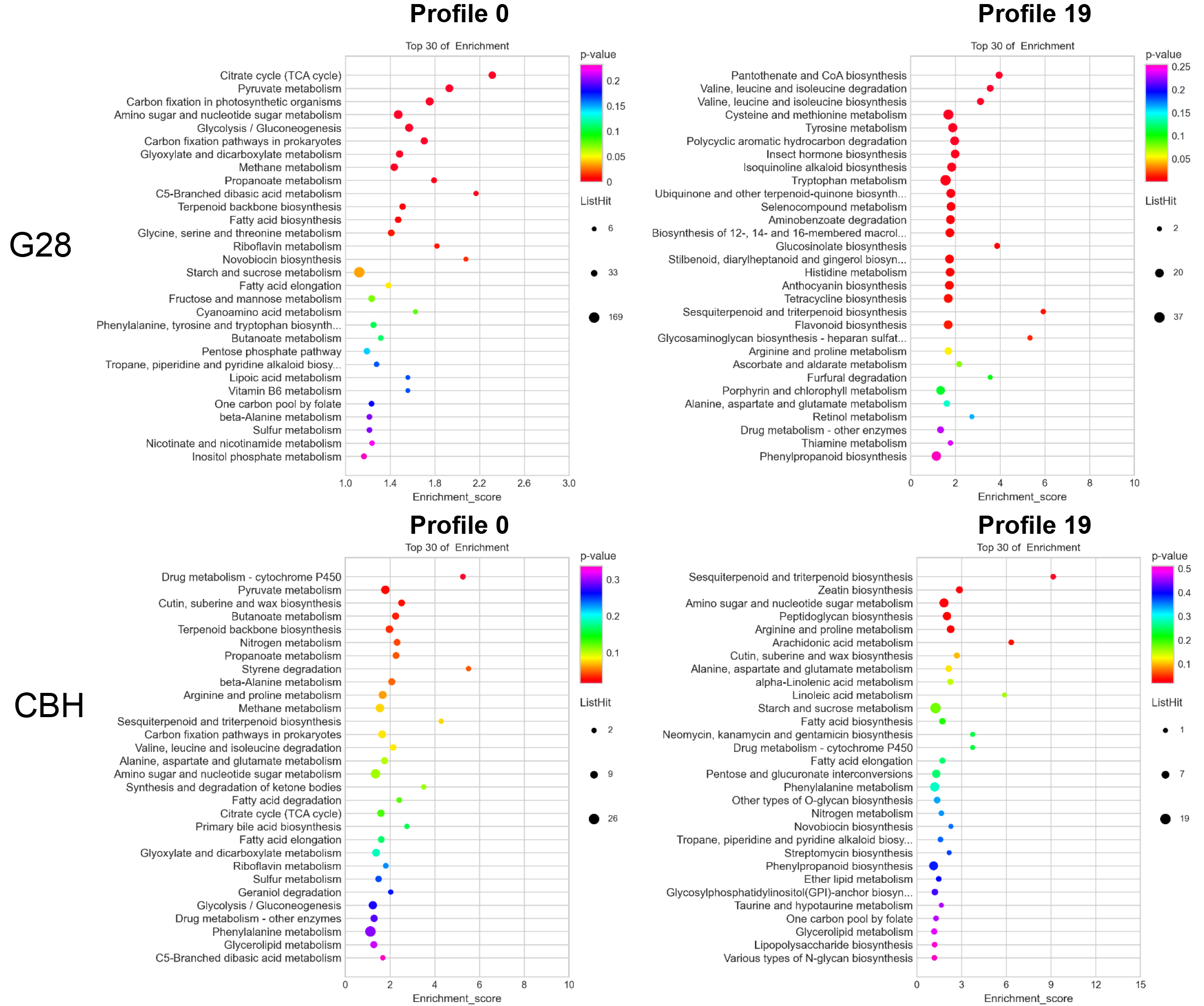

Supplement: Supplementary Figure 1 — KEGG analysis of genes in profile 0 and profile 19 of G28 and CBH. [file Image_1.jpeg]

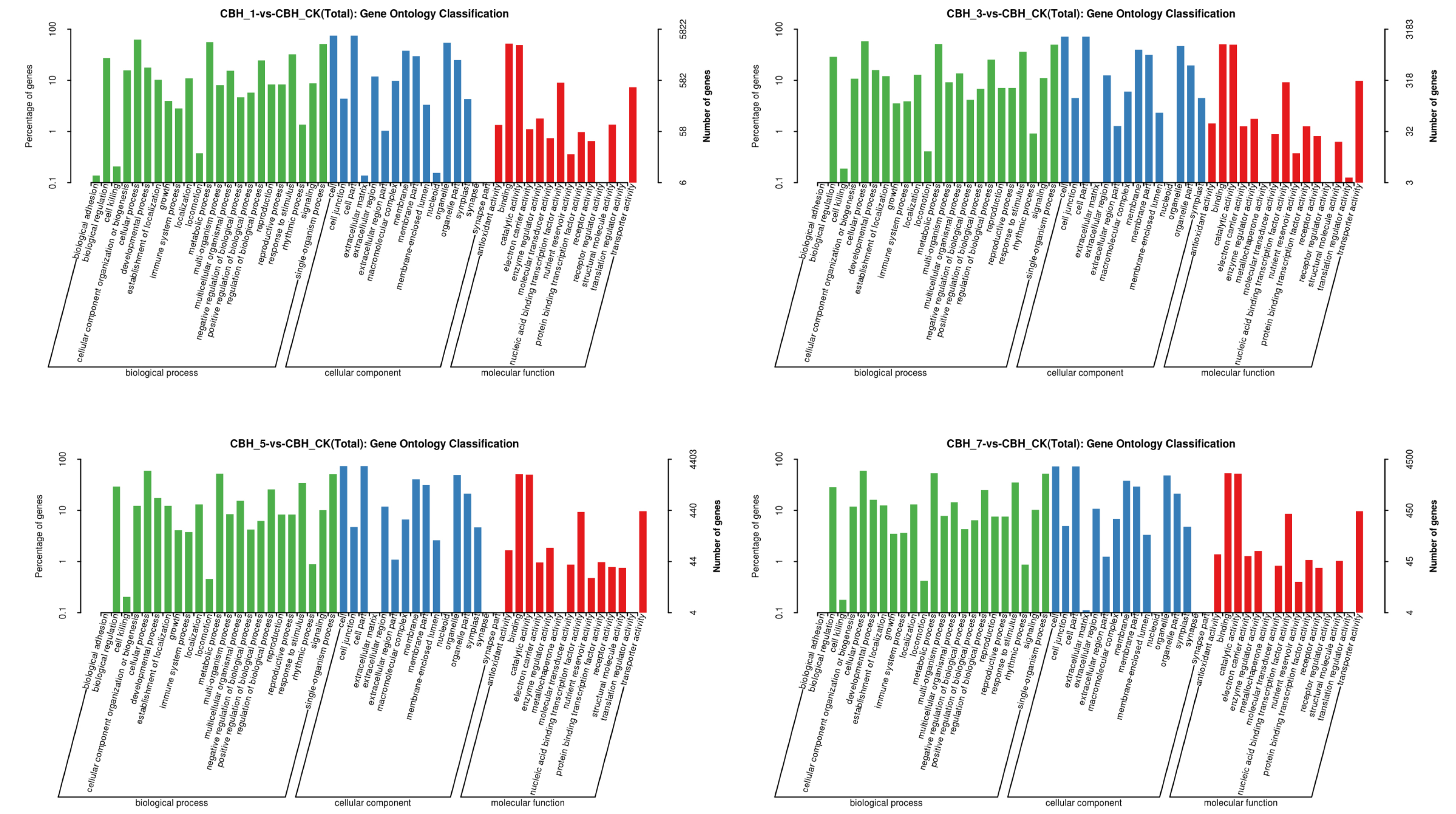

Supplement: Supplementary Figure 2 — GO enrichment analysis of DEGs in CBH after infection at 1, 3, 5, and 7 d compared with normal conditions. [file Image_2.jpeg]

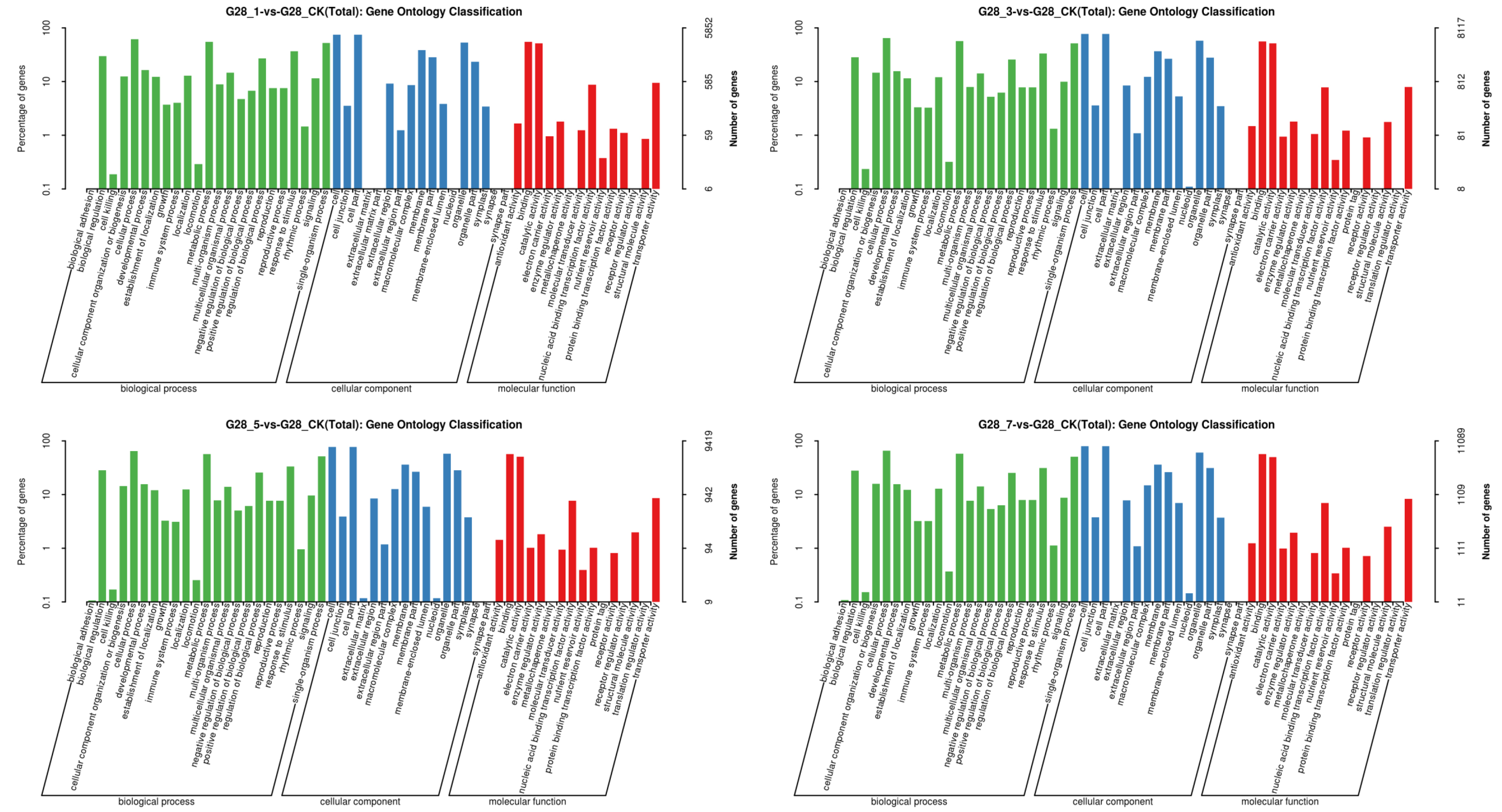

Supplement: Supplementary Figure 3 — GO enrichment analysis of DEGs in G28 after infection at 1, 3, 5, and 7 d compared with normal conditions. [file Image_3.jpeg]

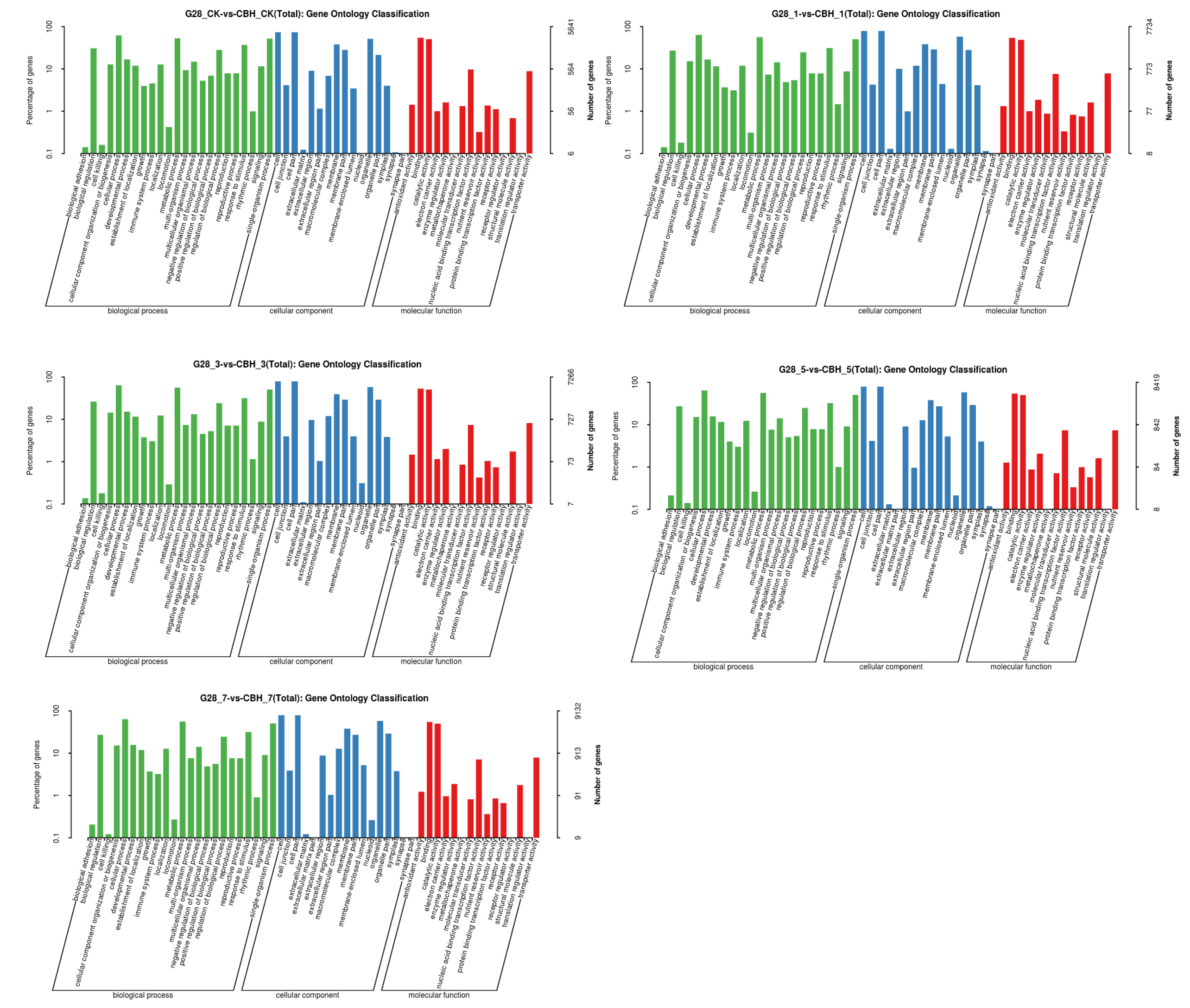

Supplement: Supplementary Figure 4 — GO enrichment analysis of DEGs between G28 and CBH at each stage after infection. [file Image_4.jpeg]

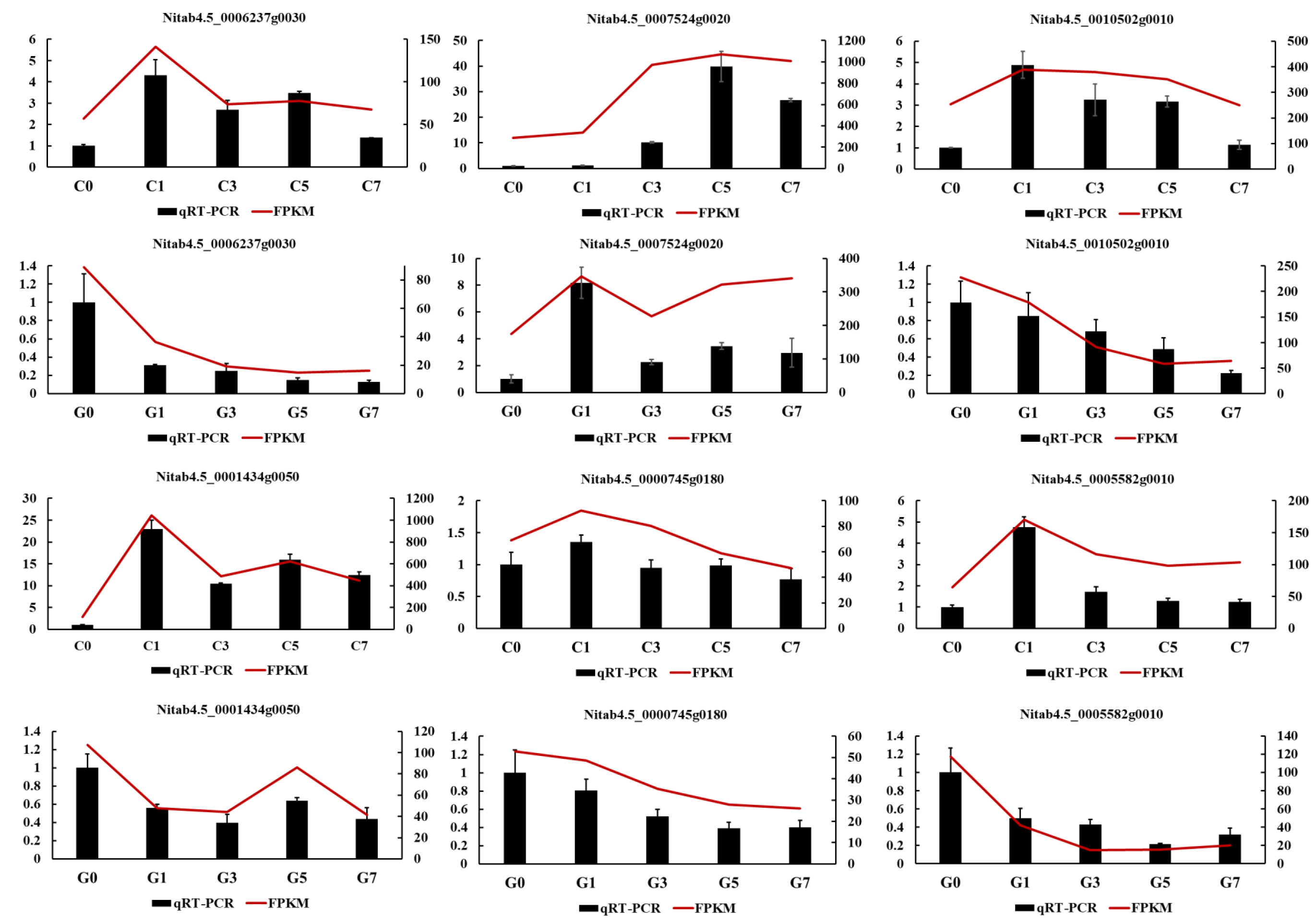

Supplement: Supplementary Figure 5 — Quantitative real-time PCR (qRT-PCR) validation and RNA-seq data. Data shown were the mean of three independent repeated experiments ± standard deviation. Error bars represent standard deviations from three independent biological replicates. [file Image_5.jpeg]
